# A Shared Neural Mechanism for Abstract Grammatical Computations across Languages in Bilinguals

**DOI:** 10.1523/JNEUROSCI.2341-25.2026

**Published:** 2026-06-15

**Authors:** Xuanyi Jessica Chen, Esti Blanco-Elorrieta

**Affiliations:** ^1^Departments of Psychology, New York University, New York, New York 10003; ^2^Neural Science, New York University, New York, New York 10003

**Keywords:** bilingualism, language production, magnetoencephalography (MEG), morphological inflection, shared syntax, unified system

## Abstract

A central question in cognitive neuroscience is how the brain implements abstract computations that must generalize across superficially different inputs. Language provides a strong test case: the same grammatical operation, such as pluralization, can be realized through distinct rules and forms across languages. Whether such transformations rely on language-specific neural systems or on abstract mechanisms that generalize across linguistic contexts remains unresolved. Crucially, these transformations must be computed online and integrated into speech planning within a tightly constrained time window. Using magnetoencephalography, we tracked the millisecond dynamics of grammatical word-form transformations during seminaturalistic phrase completion in humans of both sexes. Highly proficient Spanish–English bilinguals produced singular and plural noun forms in both languages in a design that fully orthogonalized semantic number, phonological changes, grammatical inflection, and produced language. Adjusting words to fit their grammatical context engaged a left-lateralized frontotemporal network beginning ∼100 ms after cue onset. Multivariate decoding revealed that the neural patterns supporting this computation generalized across languages, across different surface plural forms, and to pseudowords, demonstrating that abstractly equivalent operations are instantiated in the same neural substrates despite differences in linguistic form. Together, these findings provide time-resolved neural evidence for a language-general computational mechanism, showing that the brain implements grammatical transformations as abstract, generative operations. More broadly, they show how bilingualism can be used to probe general principles of neural organization, revealing how abstract computations may be shared and reused across representational systems.

## Significance Statement

Human language relies on the ability to modify words to convey information like number and tense, but languages vary widely in how these transformations are implemented. This variation raises a fundamental question in cognitive neuroscience: do such transformations depend on language-specific neural systems, or are they processed by abstract neural mechanisms that generalize across languages? We demonstrate that Spanish–English bilinguals engage a shared left frontal–temporal network when producing grammatically appropriate forms in both languages. This common neural signature emerges early during speech planning and even generalizes to novel words. These findings indicate that the brain builds abstract, reusable neural mechanisms, consistent with models where language is organized by computational principles rather than by language-specific systems.

## Introduction

Language enables humans to express infinite meanings by flexibly adapting words to fit changing contexts. This ability, known as morphological inflection, allows speakers to encode grammatical relations such as number, tense, and gender by modifying a word's form. Inflection links meaning and form, bridging lexical, syntactic, and phonological levels of representation, and offers a precise window into how the brain transforms conceptual intent into linguistic output. Yet the way grammatical relations are encoded varies widely across languages, which use distinct phonological realizations to implement different morphological rules. While English typically marks plural by adding a suffix (cat–cats), other languages may alter the internal vowel pattern (Arabic kitāb→kutub), modify the stem (German Mann→Männer), or require harmonization with vowel features of the stem (Turkish ev→evler; kitap→kitaplar). Thus, the same conceptual distinction, such as singular versus plural, can be realized through distinct morphological and phonological operations across languages.

When two languages coexist in the same mind, speakers must navigate these different systems. This raises a fundamental question: does the bilingual brain rely on a shared mechanism for inflection that operates across languages, or does each language recruit its own computational machinery? This contrast sits at the center of a major debate in bilingual neuroscience: whether the bilingual brain organizes linguistic representations by language or by universal cognitive principles that transcend linguistic boundaries.

Two major theoretical accounts make contrasting predictions. The Unified Competition Model (MacWhinney et al., 2005; MacWhinney, 2022) posits that once a mechanism is established for a given linguistic operation, it can be flexibly recruited across languages whenever the relevant structures align. In contrast, the shallow structure hypothesis (Clahsen and Felser, 2006a,b, 2018) argues that first and second languages rely on distinct neural mechanisms, even for formally similar operations. In the case of inflection, the first predicts shared mechanisms across languages with parallel morphological rules, while the second predicts language-specific systems regardless of overlap.

Empirical findings to date have been mixed. Some studies report spatially overlapping activation for morphological or syntactic processing across languages (Musso et al., 2003; Sakai et al., 2004; Perani and Abutalebi, 2005), while others find increased activation for one language over the other (Hasegawa et al., 2002; Golestani et al., 2006; Lehtonen et al., 2009; Ip et al., 2017) or overlapping but language-specific effects (Meykadeh et al., 2021; Gao et al., 2023). However, these studies have mostly focused in comprehension and have not studied planning during language generation.

**Figure 1.**
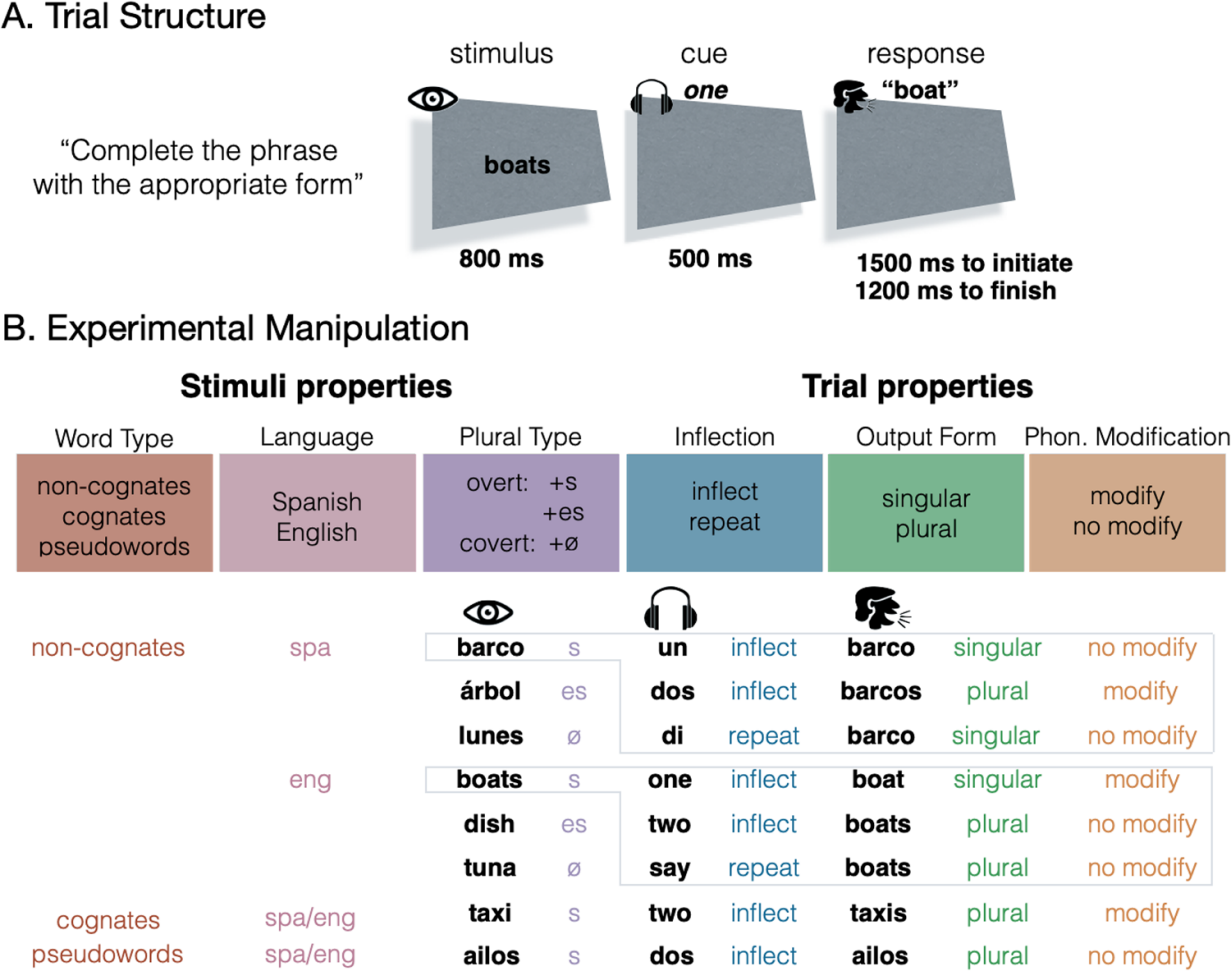
***A***, Trial structure. On each trial, participants viewed a noun on the screen for 800 ms (e.g., boats), followed by an auditory cue presented for 500 ms (e.g., *one*). The auditory cue provided the grammatical context, prompting either an inflection (*one/un*, *two/dos*) or a repetition (*say/di*). Participants then produced the appropriate form aloud (e.g., boats*, one*→“boat”), with 1,500 ms to initiate and 1,200 ms to complete the response. ***B***, Experimental manipulation. This design orthogonally manipulated three factors: Inflection (Inflect vs Repeat, denoted in blue), Output form (Singular vs Plural, in green), and Phonological Modification (Modify vs NoModify, in orange). Stimuli included noncognates, cognates, and pseudowords (Word Type, in red) in both English and Spanish (Language, in pink) and included words that use three different pluralization types Overt (*-s*, *-es*), and Covert (*ø,* in purple).

During language production, morphological operations must be planned and executed in real time and integrated with both conceptual and phonological processing. Studying inflection in production therefore poses unique challenges, as number marking entails both semantic distinctions (e.g., *one “*boat” vs *two “*boats” refer to different meanings) and phonological modifications (adding or omitting a phoneme to the noun). To isolate morphological computations from these confounds, we adopted a phrase-completion paradigm previously validated in fMRI and magnetoencephalography (MEG; Sahin et al., 2006; Shapiro et al., 2006; Willms et al., 2011; Blanco-Elorrieta et al., 2022). In this design, participants view a noun (e.g., boat) and hear an auditory cue that provides grammatical context (e.g., *one*, *two*, or *say*), requiring them to produce the corresponding form (e.g., *one* “boat,” *two* “boats,” *say* “boat”; Fig. 1). By crossing the cue type with whether the visually presented form is singular or plural, the paradigm dissociates semantic number (Plural or Singular), the requirement to perform a morphological operation (Inflect vs Repeat), and whether the phonological form is changed or remains the same as the cue (Modify vs NoModify).

We tested highly proficient Spanish–English bilinguals, two languages that mark singular–plural distinctions with identical surface realizations (+s, +es, ø). This rare alignment allows us to vary language while holding phonological form constant, providing an ideal test of whether morphological inflection depends on shared or language-specific neural mechanisms. Using a fully orthogonalized design, we aimed to characterize the neural architecture supporting inflection and to determine whether the bilingual brain organizes this process according to language membership or cross-linguistic universal principles.

## Materials and Methods

### Participants

We recruited 23 highly proficient Spanish–English bilinguals for the study [*age, 18–45 (M_Age_ = 25.7;* SD*_Age_ = 7.4);* g*ender*, *17 female*s*, 5 male*s*, 1 nonbinary*]. All participants were right-handed, reported no neurological or reading disorders, and had no metal in their body. Participants completed a Language Use Questionnaire (adapted from Kastenbaum et al., 2019), a comprehensive language background test that assesses, for each language, daily use, language of instruction at each educational level, exposure, and self-reported ability in hearing, speaking, reading, and writing across their lifespan. All participants rated their ability higher than 5 out of 7 across all four modalities and have an average ability rating of 96.4% for Spanish (SD *=* *5.4%*) and 98.4% for English (SD *=* *4.3%*). Participants' age of acquisition (AoA) was *M_AoA_Spanish_* *=* 0*.39 *years old (SD *=* *3.07*) and *M_AoA_English_* *=* *86 *years old (SD *=* *4.06*). Four reported the same AoA for the two languages, 5 reported earlier AoA in English, and 14 reported earlier AoA in Spanish. Based on a language dominance calculation (Chen and Blanco-Elorrieta, 2025), 12 participants were Spanish-dominant, 9 were English-dominant, and 2 were fully balanced. All participants provided written informed consent prior to participation. The study protocol was reviewed and approved by the New York University (NYU) Institutional Review Board (IRB), and all procedures were conducted in accordance with institutional guidelines. Participants were compensated for their time at a rate approved by the NYU IRB.

### Experimental design

#### Trial structure

Participants performed a phrase-completion task that manipulated language (Spanish vs English), morphological operation (Inflect vs Repeat), and plural type (Overt, *-s, -es*; Covert, *ø*). Each trial began with a fixation cross (300 ms), followed by a visually (presented noun, e.g., boat*,* 800 ms) and an auditory cue (e.g., *two*, 500 ms). The cue provided the start of a phrase (e.g., after seeing boat, hearing *two* prompted the response “boats”). Participants had up to 1,500 ms to initiate a verbal response and 1,200 ms to complete their response (Fig. 1).

This design adapted earlier paradigms used in fMRI and ECoG (Sahin et al., 2006, 2009; Shapiro et al., 2006; Lee et al., 2018), with one key modification (as in Blanco-Elorrieta et al., 2022): the grammatical context (*one/un, two/dos, say/di*) immediately preceded production rather than separated by the target word. We chose this format to approximate naturalistic phrase production and minimize the sense that the task involved mere word play with suffixes.

#### Task design

A central goal of our design was to isolate morphological inflection from semantic and phonological confounds. To achieve this, we orthogonalized the key dimensions of the task (Fig. 1). On the stimulus side, we varied language (Spanish vs English) and the word's pluralization type (Overt, *-s, -es*; Covert, -*ø*). On the trial design side, we varied morphological operation (Inflect vs Repeat), output form (Singular vs Plural), and phonological modification (Modify vs NoModify). This structure allowed us to separate factors that are usually confounded in natural speech. For instance, to dissociate number from phonological change, nouns were presented in either singular or plural form, and auditory cues (*one/un* or *two/dos*) required participants to adjust the form. Depending on the plural type, this sometimes required a different phonological realization (e.g., adding *-s*: boat, *two*→*“*boats”; removing *-s*: boats, *one*→*“*boat”), and sometimes it remained the same (e.g., Covert plurals: tuna*, two*→*“*tuna”; NoModify cases: boats, *two*→*“*boats”).

To dissociate semantic number from the morphological operation, we included a repeat condition with the cue *say/di*, in which participants simply repeated the word on screen (e.g., boat*, say*→*“*boat”; boats, *say*→*“*boats”). Thus, participants produced both singular and plural forms, with either the same or different phonological realizations depending on plural type, but only inflection trials required a true morphological computation. By fully crossing these dimensions, the design isolated pure inflectional processes while controlling for semantic and phonological factors.

#### Stimuli

We selected 96 nouns (48 English and 48 Spanish), divided equally across the three plural types: 16 with *-s*, 16 with -*es*, and 16 where the plural and singular share the same form (-ø). Spanish nouns were selected so that their inflectional process was strictly parallel to English: all were masculine nouns, ensuring that only number inflection varied in the task. These words are all noncognates and constitute the stimuli for our main analyses.

To complement the main analysis, we also tested cognates and pseudowords. With noncognates, any difference observed between inflecting English versus Spanish words could stem from two sources: (1) the inflectional mechanisms themselves being different across languages or (2) using different lexical entries in English and Spanish. Cognates (*N* = 16) help disambiguate these possibilities because they share the same lexical root across languages. If differences in neural responses still appeared even for cognates, where the lexical root is held constant, this would clarify that the divergence arises from the inflectional mechanisms rather than from language-specific lexical representations.

Pseudowords (*N* = 16) provided a further diagnostic. If English–Spanish overlap were observed for noncognates in our main analysis, pseudowords allowed us to test whether such overlap depends on the presence of an existing lexical entry or whether the relevant mechanisms are abstract and universal enough that they operate over any form that requires inflection. The pseudowords were constructed to follow the phonotactic and syntactic rules of both English and Spanish and were matched to the cognates for consonant–vowel structure. The same set of cognates and pseudowords was presented in both languages.

Stimuli across all conditions were matched as closely as possible for length, number of phonemes, number of syllables, and log frequency, although perfect equivalence was not possible given cross-linguistic phonological differences (i.e., Spanish words are longer in general; Table 1). Log frequency for English words was calculated combining raw frequencies of both singular and plural forms of each word extracted from SUBTLEX-US (Brysbaert and New, 2009). Log frequency for Spanish words was calculated from the lemma frequency through EsPal (Duchon et al., 2013). Both corpuses were based on movie subtitles. To minimize semantic confounds, the 16 noncognate stimuli with the *-s* plural marker in English and Spanish were translation equivalents. Before the experiment began, participants were shown the singular and plural forms of all words.

**Table 1.** Linguistic properties of the stimuli, separated by the three stimuli properties (Word Type, Plural Marker Type, and Language)

Word Type		Noncognates			Cognates
Plural marker		-s	-es	-o	-s
English	# letters	5.1 (1.7)	5.3 (1.3)	5.7 (1.7)	4.7 (0.87)
	# phonemes	4.3 (1.9)	4.4 (1.3)	4.6 (1.3)	4.6 (0.81)
	# syllables	1.4 (0.50)	1.3 (0.48)	1.6 (0.63)	2.0 (0.37)
	log frequency	0.84 (0.61)	0.88 (0.74)	0.77 (0.51)	0.61 (0.69)
Spanish	# letters	5.2 (1.7)	5.6 (0.81)	6.6 (1.4)	4.7 (0.87)
	# phonemes	4.9 (1.5)	5.5 (0.82)	6.6 (1.4)	4.6 (0.81)
	# syllables	2.3 (0.45)	2.1 (0.25)	2.5 (0.2)	2.0 (0.37)
	log frequency	0.66 (0.56)	0.69 (0.55)	0.40 (1.2)	0.63 (0.74)

#### Block design

Each noncognate word in the main analysis was presented twice in each visual × auditory prompt combination, and each cognate and pseudoword was presented once in each of its possible combinations. For nouns with an overt plural marker, there are six possible combinations: 2 visual forms [singular (boat) and plural (boats)] × 3 auditory prompts (*one/un*, *two/dos*, *say/di*). In contrast, for nouns with a covert plural marker, there are three possible combinations: 1 visual form (*tuna*) × 3 auditory prompts. This resulted in 672 trials per language.

All trials were divided into 16 single-language blocks (eight English, eight Spanish) that were presented in an interleaved order. At the start of each block, participants were given written instructions in the language of the upcoming block. Each block contained an equal distribution of stimulus and trial conditions (Word Type, Plural Type, Inflection, Output Form, and Phonological Modification), resulting in 84 single-language trials per block. The average duration of each trial was 3.2 s, resulting in blocks of 4.5 min and a total experiment duration ∼72 min. Participants were allowed to rest as long as desired between blocks.

Trial order was randomized with two constraints: the same auditory prompt could not appear more than three times consecutively and items sharing the same root were separated by at least four trials. Noncognates were presented once in each condition across the first eight blocks and once again across the latter eight blocks. Block order (English vs Spanish) and the distribution of stimuli within blocks were counterbalanced across participants.

### MEG methods

#### Data acquisition

Before MEG recording, each participant's head shape was digitized using a Polhemus dual source handheld FastSCAN laser scanner (Polhemus). Digital fiducial points were recorded at eight locations: the nasion, left and right tragus, two points anterior of the left and right tragus, and three points on the forehead. Five marker coils were placed at the same positions (excluding the nasion and tragus) to localize that head relative to the MEG sensors. The position of these markers was measured immediately before and immediately after the experiment to correct for head movement during the recording.

MEG data were collected in the KIT/NYU MEG Lab in New York using a whole-head 157 channel axial gradiometer system (Kanazawa Institute of Technology) as participants lay in a dimly lit, magnetically shielded room All written cues were displayed in arial size 40, presented foveally using the Presentation software (Neurobehavioral System), and subtended in a range from 1.65° height and 2.55° width on a screen ∼85 cm from the subject. Auditory cues were presented via MEG-compatible foam headphones. Vocal responses were captured with an MEG-compatible microphone (Shure PG 81). MEG data were sampled at 1,000 Hz with a 200 Hz low-pass filter. Environmental noise was reduced by exploiting eight magnetometer reference channels located away from the participants' head via the continuously adjusted least-squares method (Adachi et al., 2001) implemented in the MEG Laboratory software (Yokogawa Electric Corporation and Eagle Technology Corporation).

#### MEG data preprocessing

Data were preprocessed and analyzed with MNE-Python (Gramfort et al., 2013, 2014) and Eelbrain package (https://pythonhosted.org/eelbrain). Noise-reduced MEG data, the digitized head shape, and the sensor locations were imported into MNE-Python (Gramfort et al., 2014). Bad channels were identified and interpolated following the spherical spline method (Perrin et al., 1989), resulting on 8.6 interpolated channels on average. If the participant's session was split into two MEG recordings, we first aligned them using the maxwell_filter function and then concatenated the two recordings. We applied independent component analysis to our raw data to remove components corresponding to blinks, heartbeats, and motion artifacts. Subsequently, we applied a strict artifact rejection routine as in Blanco-Elorrieta and Pylkkänen (2015; 2016) and Blanco-Elorrieta et al. (2022) to individual epochs to ensure that oral and manual artifacts did not contaminate the data. Specifically, we (1) rejected all epochs that contained amplitudes >2,500 fT/cm for any sensor after noise reduction, (2) rejected any epoch that contained sudden increases in the magnitude of the signal caused by artifacts (e.g., muscular movements), and (3) applied a 40 Hz low-pass filter that eliminated any remaining movement artifacts from our data, given that the γ-frequency range (>40 Hz) is reportedly the one affected by muscle artifact contamination such as phasic contractions (Yuval-Greenberg and Deouell, 2009; Gross et al., 2013). We excluded from further analyses all trials corresponding to behavioral errors. This included trials with incorrect inflections (e.g., producing the singular form when plural was required or vice versa), trials in which participants failed to provide a response, and other morphologically inappropriate responses (e.g., producing an over-regularized plural such as *tuna*→*tunas*). Only correctly produced responses within the allotted time window were included in subsequent behavioral and neural analyses. After the epoch rejection and behavioral trial rejection process, 9.6% of trials were excluded from further analysis. For univariate analyses, trial count was equalized across conditions prior to statistical analysis. For multivariate decoding analyses, we did not equalize trial counts to maximize number of trials used for training. Instead we used a performance metric, ROC-AUC, that is robust with imbalanced trial numbers.

#### MRI acquisition and coregistration

High-resolution T1–weighted anatomical images were acquired on a 3 T Siemens Prisma scanner with a 64-channel head coil at the NYU Center for Brain Imaging. Images were collected in 240 sagittal slices with 0.9 mm isotropic voxels (TR, 2,300 ms; TE, 7.1 ms). MRI data were preprocessed with FreeSurfer's automatic reconstruction pipeline (Fischl, 2012). Surfaces of the brain, inner skull, outer skull, and scalp were generated using the watershed method (Ségonne et al., 2004) to construct a boundary element model (BEM). The scalp surface was then aligned with the digitized head shape to coregister MEG and MRI data.

#### Source localization

To transform the data from sensor to source space and evaluate where in the cortex the activity originated, MEG data were coregistered to each participant's structural MRI. An ico-4 source space was created, consisting of 2,562 potential dipole sources per hemisphere. For each source, a forward solution was computed using the BEM, which estimates the magnetic field at each MEG sensor in response to a current dipole at that location.

The low-pass filtered epochs were downsampled by 5 and baseline corrected using the 300 ms fixation period prior to visual stimulus onset (i.e., when participants had completed their previous response and were awaiting the next trial with a fixation on the screen). The inverse solution was then computed from the forward solution and the grand average activity across trials, yielding the most likely distribution of neural sources. The resulting minimum norm estimates of neural activity (Hämäläinen and Ilmoniemi, 1994) were converted into normalized noise estimates at each location to generate statistical parametric maps (SPMs), providing millisecond-level information about the statistical reliability of the signal. These maps were further transformed into dynamic maps (dSPM).

To assess the spatial resolution of these maps, we computed the point-spread function for multiple cortical locations, which characterizes the spatial blurring of the true activity in the reconstructed maps and provides an estimate of brain activity with the highest possible spatial and temporal accuracy (Dale et al., 2000). The inverse solution was applied to each trial using a free orientation approach, which models dipole currents without restricting dipole orientation. For vector summarization, pooling is performed by taking only the norm of the free orientations.

### Statistical analyses

#### Behavioral analyses

We analyzed the accuracy and reaction times (RTs). Participants' verbal productions and accuracy were coded by a highly proficient Spanish–English bilingual research assistant. RTs were obtained from the onset of participants' verbal production. Only correct trials were included in the RTs analyses and the MEG analyses. For one participant, we excluded six blocks (three English, three Spanish) from both behavioral and MEG analyses due to below threshold accuracy in those blocks (<80%). We used the rmAnova function in the statsmodels package in Python to administer all the repeated-measures ANOVAs and the stats module in the scipy package for the paired *t* tests. Behavioral analyses were conducted in three stages, corresponding to the structure of the experimental design.

1. *Morphological and language factors (noncognate items only).* To assess the effects of morphological demands independently of lexical overlap, we first restricted analyses to noncognate real words. Repeated-measures ANOVAs were conducted on accuracy and RTs with the following within-subject factors: Inflection (Inflect vs Repeat), Language (English vs Spanish), and Plural Marker Type (Overt vs Covert). Plural Marker Type captured whether that lexical item required its plural marking to be phonologically realized (e.g., overt suffix) or not (e.g., covert inflection).
2. *Phonological modification effects (Inflect trials, overt markers only).* To isolate phonological realization demands, we further analyzed Inflect trials involving overt plural markers. Within this subset, we compared items that required a phonological modification of the stem (Modify) to those that preserved the input form (NoModify) using paired two-tailed *t* tests. To further understand whether phonological modification incurs disproportionate costs for singularization over pluralization, we conducted repeated-measures ANOVAs with the following within-subject factors: Language (English vs Spanish), Phonological Modification (Modify vs NoModify), and Output Form of the verbal production (Singular vs Plural).
3. *Lexical status effects (Word Type).* To assess the contribution of lexical overlap to the sharedness of inflection mechanisms, we conducted separate repeated-measures ANOVAs crossing: Language (English vs Spanish), Word Type (Cognate, Noncognate, Pseudoword) and Inflection (Inflect vs Repeat). Planned pairwise comparisons were performed using two-tailed paired *t* tests where appropriate.

#### Source-localized univariate analyses

We analyzed source-localized current estimates using nonparametric temporal and spatiotemporal cluster tests in the Eelbrain package (https://pythonhosted.org/eelbrain). Because planning for oral responses began as soon as participants heard the auditory cue, we focused our analyses on the time window immediately following auditory onset and extending until 100 ms after audio offset (600 ms after audio onset), which also ensured that neural measures were uncontaminated by articulatory movement. Based on previous work with the same paradigm that reported frontotemporal involvement during morphological inflection (Sahin, Pinker, and Halgren, 2006; Willms et al., 2011; Blanco-Elorrieta et al., 2022), we focused on two ROIs: a left frontal region spanning Brodmann's areas 9, 10, 11, 44, 45, and 47 as defined in PALS_B12_Brodmann parcellation and a left temporal lobe ROI as defined in PALS_B12_Lobes Parcellation in MNE.

For ROI-specific temporal cluster analyses, we first computed mean current estimates across all sources within each ROI. Tests at each time point were thresholded at *p* < 0.05 (uncorrected), and clusters were identified if they contained at least 25 contiguous time points exceeding this threshold. For each surviving cluster, *F* values were summed to form a cluster-level statistic. Statistical significance was assessed using 10,000 permutations of the data (Maris and Oostenveld, 2007). In each permutation, we randomly shuffled the condition labels and recomputed the cluster statistic, yielding a null distribution of cluster-level *F* values against which the observed cluster statistics were evaluated. We conducted post hoc comparisons between conditions within significant clusters using paired-sample *t* tests, with false discovery rate (FDR) correction applied across tests (Benjamini and Hochberg, 1995).

For spatiotemporal clustering analyses, we computed maps of *F* values across both sources and time points within each ROI. These maps were thresholded at *p* < 0.05 (uncorrected), and clusters were defined as contiguous regions in space and time exceeding this threshold. Clusters were retained if they lasted at least 25 ms and included a minimum of 10 vertices. As above, cluster-level statistics were computed as the sum of *F* values within each cluster, and statistical significance was assessed using the same permutation procedure (10,000 permutations; Maris and Oostenveld, 2007).

*Main analysis*. Our main analyses focused on noncognate real words. For each ROI, we extracted the mean current estimate across sources and submitted it to a 2 × 2 × 2 ANOVA with the factors Morphological Inflection (Inflect vs Repeat), Plural Marker Type (Overt +s/+es vs Covert ø), and Language (English vs Spanish). This omnibus analysis assessed whether the magnitude of the inflection effect depended on the language of production, independent of the surface form that it takes, thereby testing for global cross-language differences in inflectional processing. Because Inflect trials necessarily require a grammatical transformation whereas Repeat trials do not, Inflect > Repeat contrasts may reflect a combination of inflection-specific computation and broader task demands (e.g., increased difficulty). Accordingly, beyond these omnibus contrasts, we place particular emphasis on analyses that test the inflectional contrast while varying surface form and/or maintaining relative task difficulty (e.g., cross-language decoding, overt-to-covert generalization, and cross-output form generalization). These tests provide a more selective diagnostic of inflectional processing because successful generalization requires a shared representational format across contexts that differ in perceptual and production demands.

*Dissociating morphological inflection from phonological modification.* Next, to separate the effects of performing a morphological operation from the effects of phonological modification, we restricted analyses to trials with overt plural markers (+s/+es). Covert plurals (ø) never change the phonological shape of the word and therefore cannot distinguish whether neural differences arise from inflection itself or from phonological modification. Overt plural markers (+s/+es), in contrast, include both cases where the form changes (Modify) and cases where it does not (NoModify), enabling this dissociation. Within this subset, Inflection (Inflect vs Repeat) and Phonological Modification (Modify vs NoModify) are not fully crossed: Repeat trials inherently involve no phonological change, making the Repeat-Modify cell structurally impossible. Consequently, these factors collapse into three meaningful empirical conditions: Inflect-Modify, Inflect-NoModify, and Repeat. We therefore ran a second series of ANOVAs using this three-level condition as a factor, along with Language (English vs Spanish) and Output Form (Singular vs Plural). This analysis tested whether neural responses to inflection depend on phonological modification and whether this dependence differs across languages.

*Identifying the spatiotemporal profile of inflection and overlap in each language*. To determine the neural locus and time course of morphological inflection within each language, we next conducted separate spatiotemporal cluster tests for English and Spanish. Within each language, Inflect and Repeat trials were contrasted while controlling for plural marker type. These within-language analyses allowed us to identify the regions and time windows sensitive to inflection independently in English and in Spanish. Then, to directly test whether English and Spanish recruit overlapping neural mechanisms for inflection, we compared the significant clusters identified in the two within-language analyses. Spatiotemporally overlapping clusters would indicate that the same neural populations support inflection in both languages, consistent with the Unified Competition Model, which predicts shared mechanisms for formally parallel morphological operations. Conversely, nonoverlapping clusters would support the shallow structure hypothesis, which proposes language-specific mechanisms even when operations are structurally similar.

*Assessing the role of word type in shared inflectional processing*. In addition to the primary analyses on noncognate words, we examined whether cross-language overlap in inflection depended on word type (noncognates, cognates, pseudowords) using temporal cluster test on the mean current estimates for each ROI. Cognates allowed us to test whether shared inflectional machinery requires a shared lexical root across languages. If shared inflectional effects were driven by lexical alignment, cognates should show greater cross-language overlap than noncognates. Conversely, if inflectional computation is abstracted from lexical identity, cognates and noncognates should pattern similarly. Pseudowords provided a complementary diagnostic: if pseudowords patterned like real words and showed overlapping neural bases of inflection across languages, this would indicate that the machinery supporting inflection is sufficiently abstract and language-general to operate without an existing lexical entry.

#### Sensor-level multivariate analyses

While univariate analyses can detect amplitude differences between conditions, they are insensitive to distributed patterns of activity that may encode meaningful information even when overall signal magnitude is similar. To assess whether inflection is represented in spatially distributed neural patterns, we conducted a multivariate pattern analysis at the sensor level.

At each time point, we trained a logistic regression classifier to discriminate between two trial types using the full pattern of sensor activity. To characterize the temporal dynamics of the information carried by these patterns, we computed temporal generalization matrices (King and Dehaene, 2014): after training a classifier at time *t*, we tested its ability to discriminate between the same two trial types at all other time points *t′*. This procedure reveals whether the neural representation that supports the classification is transient, sustained, or reactivated over time and was applied in all decoding analyses described below. We used this decoding framework in two ways:

*Within-condition decoding*. First, we asked whether inflection is decodable within specific task contexts. For example, we trained a classifier to distinguish Inflect versus Repeat trials within English, or within Spanish, using the pattern of sensor activity at each time point and its temporal generalization. Successful within-condition decoding indicates that the neural signal in that context carries information that reliably distinguishes the two trial types.

*Cross-condition decoding*. Second, we tested whether the neural representation of inflection generalizes across contexts. In cross-condition decoding, a classifier trained in one condition is tested on independent data from another condition—for instance, training on Inflect versus Repeat in English and testing on Inflect versus Repeat in Spanish. Above-chance cross-condition decoding indicates that the same or highly similar representational format supports inflection across those contexts, whereas failure to generalize suggests condition-specific neural codes.

We applied this decoding approach across different Languages (English vs Spanish), Plural Types (Overt vs Covert), Output Forms (Singular vs Plural), and Word Types (noncognates, cognates, pseudowords). All classification analyses were conducted within subjects using MNE-Python and scikit-learn. All 157 MEG channels were used as features, and no dimensionality reduction was applied. Classification performance was evaluated using the ROC-AUC metric, which is a robust metric for analyses with imbalanced trial numbers. For each participant, ROC-AUC scores were averaged across trials at each time point. Group-level statistics were obtained by contrasting mean AUC values against chance (0.5) using one-sample one–tailed *t* tests, with FDR correction across time points (Benjamini and Hochberg, 1995). Reliable decoding was defined as at least 25 ms consecutive trained and tested times with performance significantly above chance at *p* < 0.05 (corrected).

## Results

### Behavioral

Participants achieved a high overall accuracy (*M* = 94.6%; SD = 2.7%), confirming that the task was well understood and reliably performed. Only correct trials were included in RT analyses and subsequent MEG analyses.

Accuracy and RT reflected both task demands and linguistic properties of the stimuli. We first focused on noncognate words, with ANOVAs crossing Inflection (Inflect vs Repeat), Language (English, Spanish), and Plural Marker Type (Overt vs Covert). Expectedly, participants were more accurate in Repeat (*M* = 97.2%; SD = 1.9%) than Inflect trials (*M* = 93.7%; SD = 3.4%; *F* = 43.451; *p* < 0.001) and for Overt (*M* = 96.6%; SD = 2.2%) compared with Covert plural markers (*M* = 88.0%; SD = 7.9%; *F* = 26.490; *p* < 0.001). These factors interacted (*F* = 39.856; *p* < 0.001): the Inflection-related accuracy cost occurred only for Covert markers (*M*_Inflect_* = *83.0%; SD_Inflec*t*_* = *11.5% vs *M*_Repeat_* = *98.1%; SD_Repeat_* = *2.5%; *t* = −6.535; *p* < 0.001), with no difference for Overt markers (*M*_Inflect_* = *96.4%; SD_Inflect_* = *2.4% vs *M*_Repeat_* = *97.0%; SD_Repeat_* = *2.0%; *t* = −1.482; *p* = 0.153). For RT, participants were faster in Inflect trials (*M* = 909.1 ms; SD = 117.4 ms) than Repeat trials (*M* = 945.0 ms; SD = 114.1 ms; *F* = 21.708; *p* < 0.001) and for Overt (*M* = 916.6 ms; SD = 115.0 ms) compared with Covert plural markers (*M* = 942.7 ms; SD = 118.7 ms; *F* = 26.964; *p* < 0.001). These factors also interacted (*F* = 12.394; *p* = 0.002): the effect of Inflection on RT only occurred for Overt markers (*M*_Inflect_* = *902.6 ms; SD_Inflect_* = *116.7 ms vs *M*_Repeat_* = *944.1 ms; SD_Repeat_* = *114.4 ms; *t* = −7.105; *p* < 0.001) and not for Covert markers (*M*_Inflect_* = *940.0 ms; SD_Inflect_* = *122.9 ms vs *M*_Repeat_* = *948.1 ms; SD_Repeat_* = *117.0 ms; *t* = −0.961; *p* = 0.347).

Within Inflect trials with Overt markers, items requiring phonological modification were less accurate than those preserving the input form (*M*_Inflect_Modify_* = *94.7%; SD_Inflect_Modify_* = *3.6% vs *M*_Inflect_NoModify_* = *98.2%; SD_Inflect_NoModify_* = *1.6%; *t* = −6.490; *p* < 0.001), but there was no RT difference (*M*_Inflect_Modify_* = *904.2 ms; SD_Inflect_Modify_* = *119.1 ms vs *M*_Inflect_NoModify_* = *901.0 ms; SD_Inflect_NoModify_* = *115.3 ms; *t* = 0.734; *p* = 0.470). To directly assess whether singularization (plural visual form→singular output) carries a disproportionate behavioral cost relative to pluralization (singular visual form→plural output), we tested the interaction between Output Form (Singular vs Plural) and Phonological Modification (Modify vs NoModify) within Inflect trials with overt markers. This analysis revealed no significant interaction between Output Form and Phonological Modification in accuracy (*F* = 4.376; *p* = 0.05) or RT (*F* = 3.330; *p* = 0.082), indicating that singularization was not more difficult and did not incur greater behavioral cost as compared with pluralization. Thus, although phonological modification reduced accuracy overall, we did not observe evidence that singularization specifically imposes a greater response cost.

Participants were more accurate in Spanish (*M* = 95.6%; SD = 2.7%) than in English (*M* = 94.2%; SD = 3.2%; *F* = 4.684; *p* = 0.042) and responded faster in Spanish (*M* = 886.0 ms; SD = 111.7 ms) than in English (*M* = 957.0 ms; SD = 124.0 ms; *F* = 43.641; *p* < 0.001).

We next assessed the role of lexical representation, and we found that lexical status elicited a graded pattern. Accuracy was similar for real words (i.e., cognates and noncognates; *M*_cognate_* = *96.7%; SD_cognate_* = *2.6% vs *M*_non-cognate_* = *96.4%; SD_non-cognate_* = *2.4%; *t* = 0.868; *p* = 0.395), but dropped for pseudowords (*M*_pseudo_* = *91.3%; SD_pseudo_* = *5.0%; *t*s > 5.807; *p*s < 0.001). Response times, in contrast, showed a more sensitive gradient: cognates were slower than noncognates (*M*_cognate_* = *935.6 ms; SD_cognate_* = *113.9 ms vs *M*_non-cognate_* = *919.1 ms; SD_non-cognate_* = *114.0 ms; *t* = −3.385; *p* = 0.003), and pseudowords elicited the longest response times overall (*M*_pseudo_* = *985.2 ms; SD_pseudo_* = *120.6 ms; *t*s < −5.048; *p*s < 0.001). In addition, the ANOVAs further revealed a Word Type **×** Inflection interaction for both accuracy (*F* = 28.033; *p* < 0.001) and RT (*F* = 8.892; *p* < 0.001): only pseudowords showed reduced accuracy in Inflect relative to Repeat trials (*M*_Inflect_* = *89.0%; SD_Inflect_* = *6.5% vs *M*_Repeat_* = *95.9%; SD_Repeat_* = *3.9%; *t* = −5.472; *p* < 0.001), and only pseudowords showed no difference in RT for Inflect compared with Repeat trials (*M*_Inflect_* = *982.7 ms; SD_Inflect_* = *135.6 ms vs *M*_Repeat_* = *991.0 ms; SD_Repeat_* = *103.9 ms *t* = −0.627; *p* = 0.537).

Overall, despite near-ceiling performance, participants showed systematic sensitivity to inflectional demands, phonological realization, and lexical status. Inflection was selectively impaired for covert morphology and pseudowords, while language differences emerged primarily in processing speed, with participants being faster in Spanish.

### MEG results

#### Morphological inflection is shared across languages and plural markers

Our central question was whether morphological inflection engages shared neural mechanisms across languages. To test this, we first needed to establish whether inflection is represented abstractly enough within a language to generalize across different plural markers. If the neural machinery of inflection were tied to a single surface form (e.g., *-s*), any apparent overlap across languages could simply reflect phonological similarity rather than a truly abstract inflectional process. Demonstrating that inflection generalizes across overt (*-s*; *-es*) and covert markers (*ø*) therefore provides a critical prerequisite: it identifies a candidate neural mechanism abstract enough to plausibly extend across languages.

**Figure 2.**
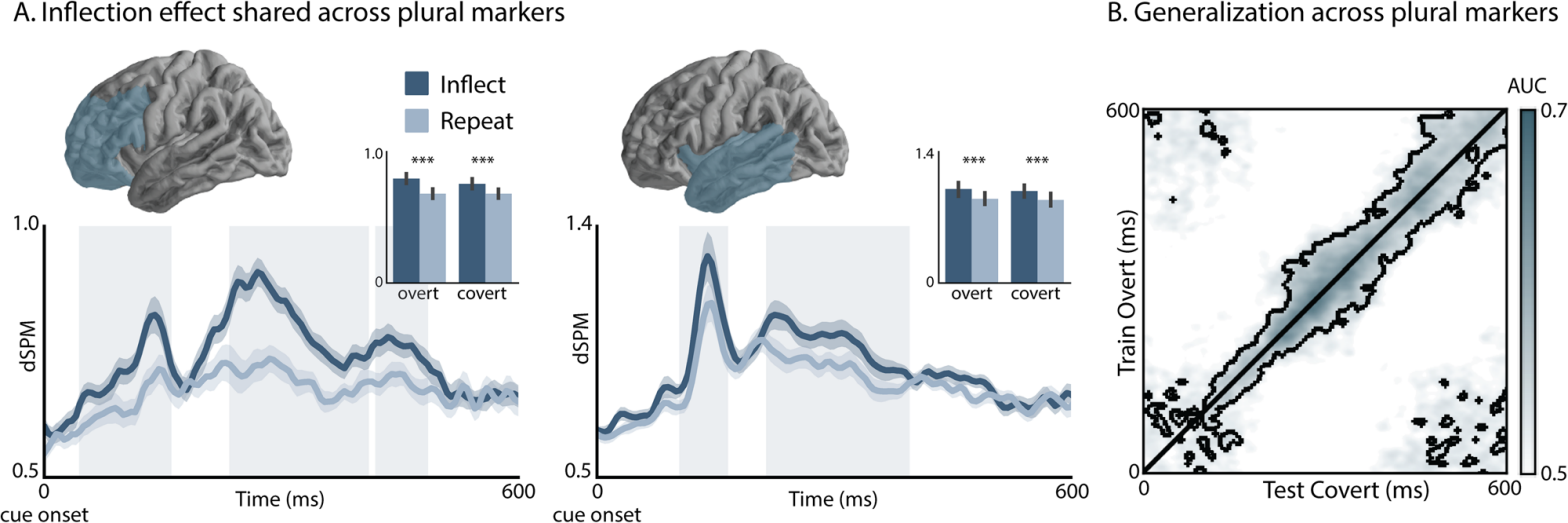
***A***, Effect of shared morphological inflection across plural markers. The brain plots show the analysis ROIs, and the waveforms show the time course of activity for Inflect and Repeat trials in dSPM. Significance time windows at *p* < 0.05 are marked with vertical shaded boxes. The bar plots show the averaged activity for all time points within the significant temporal clusters. Asterisks denote the pairwise significance of Inflect versus Repeat trials divided by Overt and Covert markers. The effect of Inflection was highly significant for both Overt and Covert markers in both ROIs (*p* < 0.001). ***B***, Decoding accuracy across time when classifiers trained on words with Overt markers are tested on words with Covert markers. Outlines mark time-generalized points where decoding exceeded chance. The temporal generalization matrix shows successful decoding along the temporal diagonal, reflecting a stable time course of inflection-related activity across Overt and Covert markers.

Using noncognate words, an ANOVA crossing Inflection (Inflect vs Repeat), Language (English, Spanish), and Plural Marker Type (Covert vs Overt) revealed robust main effects of Inflection in the left frontal ROI (45–160 ms, *p* < 0.001; 235–410 ms, *p* < 0.001; 420–485 ms, *p* = 0.015; Fig. 2*A*, left) and in the left temporal lobe (105–165 ms and 215–395 ms, both *p* < 0.001; Fig. 2*A*, right). It is important to note that cluster-based temporal statistics can yield extended significant windows when condition differences span adjacent processing stages. Accordingly, these broad temporal clusters should not be interpreted as reflecting a single sustained process, but rather contiguous timepoints over which the Inflect-Repeat contrast is reliably expressed. Importantly, we found no correlation between individual differences in the effect sizes of Inflection and the RT differences for Inflection (frontal ROI, Cluster 1, *r* = −0.044; *p* = 0.841; Cluster 2, *r* = −0.293; *p* = 0.174; Cluster 3, *r* = −0.188; *p* = 0.389; temporal ROI, Cluster 1, *r* = −0.206; *p* = 0.346; Cluster 2: *r* = −0.271; *p* = 0.211), suggesting that the neural effects for Inflection we observed are unlikely to be capturing behavioral difficulty.

Crucially, these inflection effects were shared across plural types, including those nouns that did not have overt pluralization (i.e., ø suffix plurals like *tuna*). Decoding analyses converged with this result: classifiers trained to discriminate Inflect versus Repeat trials on overt pluralization generalized successfully to covert plurals within the same language (Fig. 2*B*). This demonstrates that morphological inflection is represented by a neural process abstract enough to operate independently of surface form, identifying a candidate mechanism that could plausibly extend across languages.

Having established that this left frontotemporal response pattern supports *abstract* inflection (independent of phonological realization), we next turned toward our main question and asked whether the same inflection mechanism is engaged across languages. A simple Inflection × Language interaction in a spatiotemporal ANOVA would not answer this question: such an analysis tests whether the magnitude of the inflection effect differs between English and Spanish, but even a null interaction cannot provide positive evidence that both languages rely on the same spatiotemporal neural mechanism. To directly test sharedness, we therefore examined (1) whether the same cortical sources show inflection effects in both languages and (2) whether the same multivariate patterns support the Inflect-Repeat contrast across languages.

**Figure 3.**
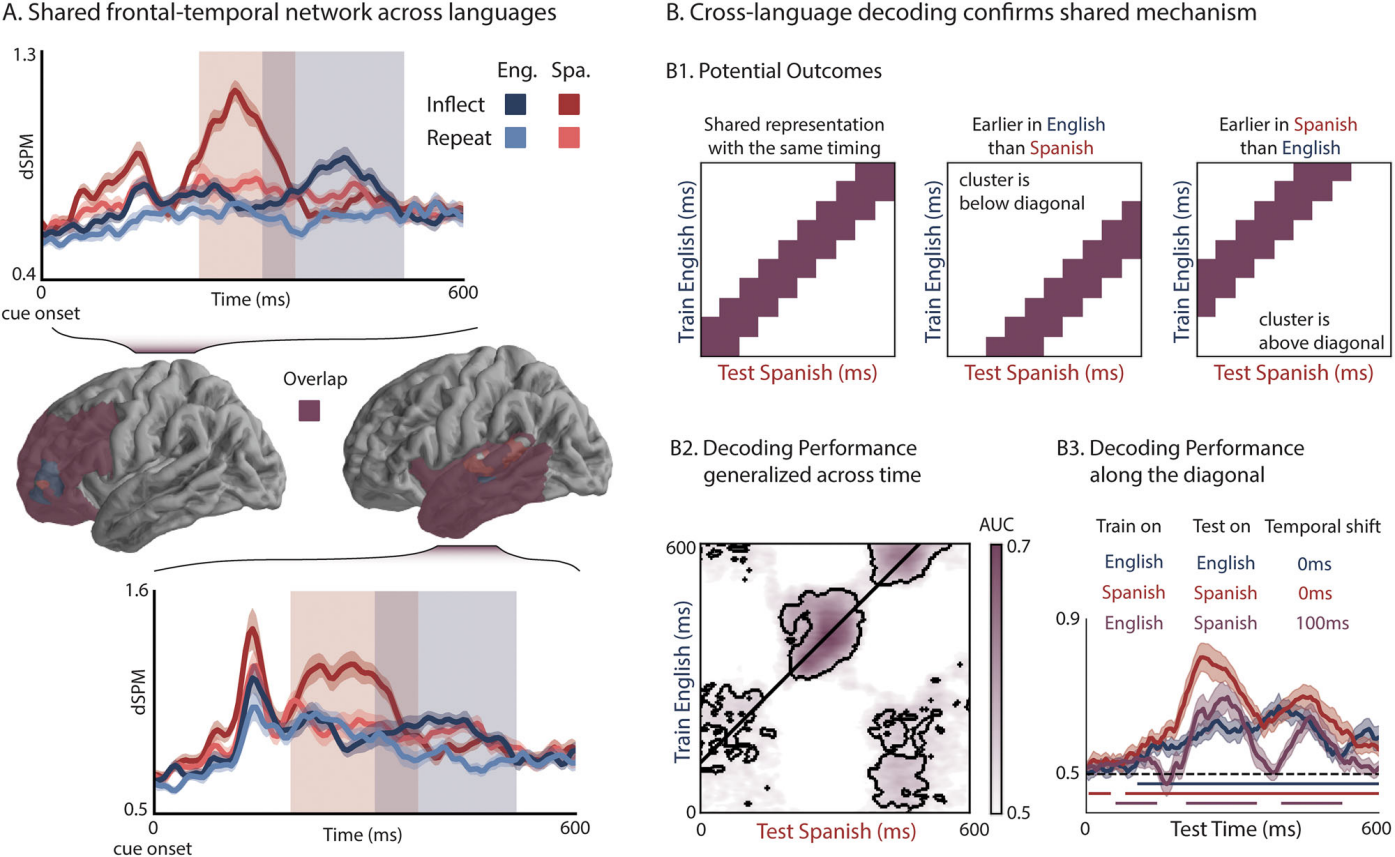
***A***, Brain plots show the spatial extent of the inflection effect for English and Spanish. Overlap reflects the shared frontal–temporal sources engaged across languages. The line plots display the time course of activity for Inflect and Repeat trials (dSPM) in each language. Significant time windows at *p* < 0.05 are shaded. ***B*1**, Schematic illustrates potential outcomes for successful cross-language decoding and how they would manifest in the train-English/test-Spanish matrix under different temporal alignment hypotheses. ***B*2**, Decoding accuracy across time when classifiers trained on English are tested on Spanish. Outlines mark time points where decoding exceeded chance. The solid line marks the optimal train–test temporal alignment for cross-language decoding. ***B*3**, Decoding performance extracted along the optimal train–test alignment defined in ***B*2**. Robust above-chance cross-decoding indicates that English and Spanish share a common signature for inflection. Significant windows (*p* < 0.001) are illustrated with horizontal bars.

Separate spatiotemporal ANOVAs in English and Spanish revealed robust inflection effects in both frontal and temporal ROIs, with largely overlapping spatial distributions (Fig. 3*A*). The key difference between languages was temporal: inflection effects emerged earlier for Spanish (frontal ROI, 70–165 ms, *p* = 0.022; 225–360 ms, *p* < 0.001; Fig. 3*A*, top; temporal ROI, 75–165 ms, *p* = 0.01; 195–375 ms, *p* < 0.001; Fig. 3*A*, bottom) than for English (60–175 ms, *p* = 0.016, in frontal ROI and 315–515 ms, *p* < 0.001, in both frontal and temporal ROIs). To quantify the spatial correspondence between languages, we computed the Jaccard index between the most robust inflection clusters in English and Spanish within each ROI (0, no overlap; 1, complete overlap). Both ROIs showed high overlap (frontal, 0.94; temporal, 0.91), indicating that inflection engages highly similar cortical populations in both languages.

Multivariate decoding analyses converged with this conclusion. Classifiers trained to discriminate Inflect versus Repeat in one language reliably decoded the same contrast in the other (Fig. 3*B*), demonstrating that the underlying neural patterns generalize across languages. Notably, peak cross-language decoding showed a consistent ∼110 ms temporal offset (Spanish→English later; English→Spanish earlier) rather than aligning along the identity line. This offset mirrors the timing differences observed in the univariate analyses and the behavioral RT advantage for Spanish, although the individual-level RT and decoding-shift measures were not correlated.

Taken together, these findings provide converging univariate and multivariate evidence that morphological inflection is supported by a shared left frontotemporal mechanism that abstracts away from surface form within a language and generalizes across languages. The temporal offset between Spanish and English reflects processing speed differences rather than distinct neural machinery, providing strong evidence for a common neural architecture for inflectional processing across languages. Importantly, while univariate Inflect > Repeat contrasts may reflect a mixture of inflection-specific computation and broader task demands, the cross-language and cross-plural–type decoding results provide a more selective diagnostic of inflectional representation. Successful generalization requires that inflection be encoded in a shared neural format that is preserved across contexts that differ in surface realization and production demands, making a purely difficulty-based explanation unlikely.

#### The morphological inflection effect is not sensitive to phonological modification

**Figure 4.**
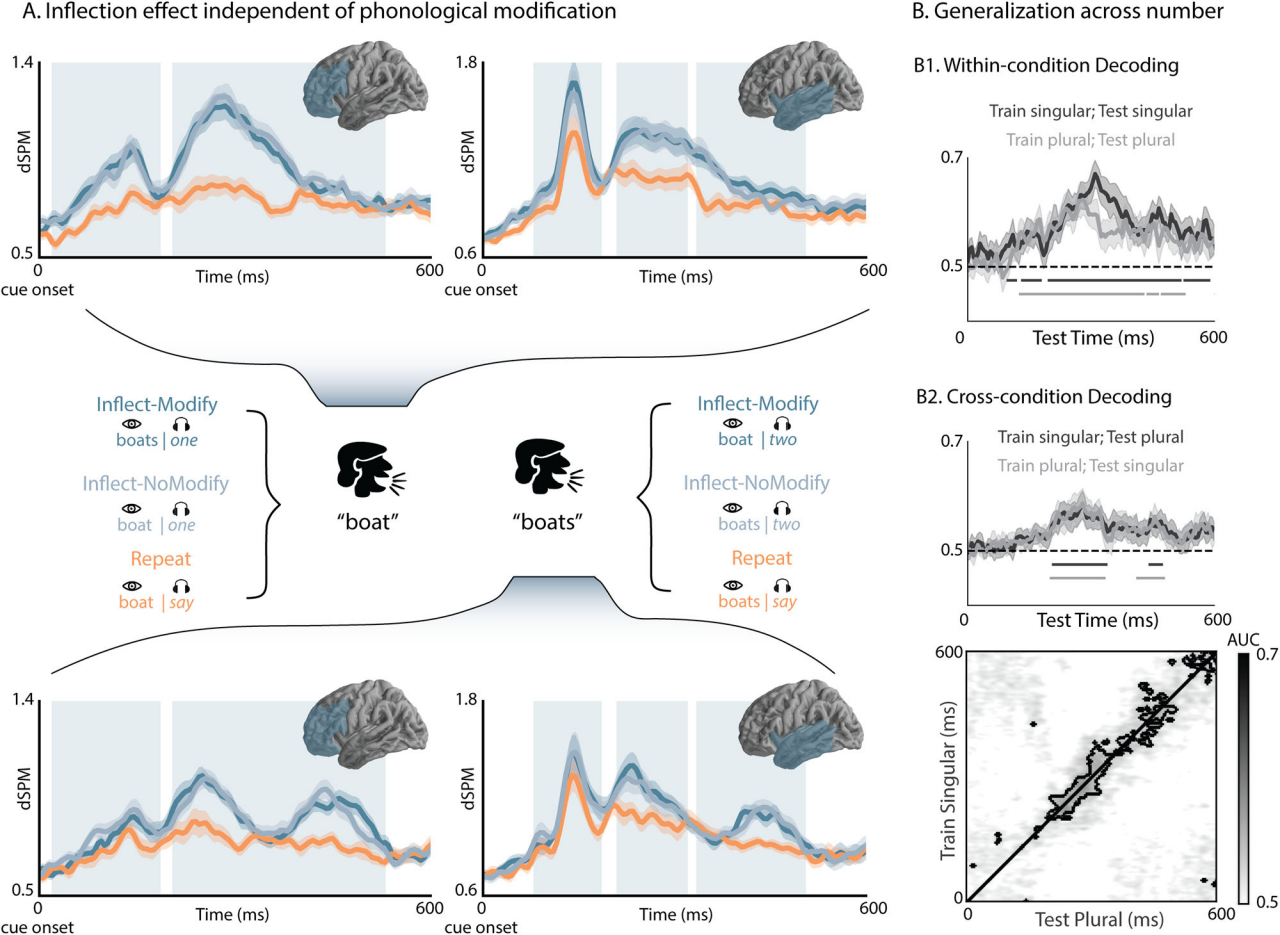
***A***, Inflection effect independent of phonological modification. Brain schematics indicate the ROIs in which the MEG analyses were conducted. The line plots show the time course of activity (dSPM) for Inflect-Modify, Inflect-NoModify, and Repeat trials. Significant time windows at *p* < 0.05 are shaded. The central schematic illustrates the structure of the Modify versus NoModify conditions. Inflection evoked strong, sustained responses in both ROIs regardless of whether overt phonological modification occurred. ***B***, Generalization across number. ***B*1**, Within-condition decoding. Performance of classifiers trained and tested to decode inflection on singular and plural items. ***B*2**, Cross-condition decoding. Performance of classifiers trained to decode inflection on singular and tested on plural items or vice versa. ***B*3**, Time-generalized decoding. The temporal generalization matrix shows successful decoding along the temporal diagonal, reflecting a stable time course of inflection-related activity across singular and plural forms.

A potential confound in identifying the neural basis of morphological inflection is that Inflect trials often involve a phonological form change (e.g., boat→*“*boats”), whereas Repeat trials never do. To test whether the clusters identified above were driven by phonological modification rather than inflection per se, we restricted analyses to trials with overt plural markers and contrasted three conditions: Inflect-Modify (inflection with phonological change), Inflect-NoModify (inflection without phonological change), and Repeat. If the previously identified clusters were merely due to phonological form changes, we would expect Inflect-NoModify to pattern with Repeat. In contrast, if the identified clusters reflected abstract morphological inflection, we should observe similar activity for Inflect-Modify and Inflect-NoModify as compared with Repeat. The results supported the latter: both Inflect conditions showed robust activation relative to Repeat, with frontal effects from 20 to 185 ms (*p* < 0.001) and from 205 to 530 ms (*p* < 0.001) and temporal effects from 80 to 185 ms (*p* < 0.001), from 210 to 320 ms (*p* < 0.001), and from 335 to 505 ms (*p* < 0.001) after auditory onset (Fig. 4*A*), indicating that the inflection effect identified here is not reducible to phonological change (Fig. 4*A*).

Next, we asked whether different phonological changes [i.e., adding a phoneme (singular→plural) vs subtracting a phoneme (plural→singular)] engage distinct processes. If addition and subtraction of phonemes were handled by distinct mechanisms, Inflect-Modify trials ending in plural (addition) should differ from those ending in singular (subtraction). No such interaction was observed. Instead, we found that producing inflected singular outputs elicited greater activation than plural outputs, whether they involved a phonological modification or not. This indicates that the inflection effect is not explained by the type of phonological change (addition vs subtraction), though inflecting singular forms appears to be more demanding than plural forms.

Decoding analyses further confirmed that the effects of morphological inflection were not a product of phonological modification. A classifier trained to distinguish Inflect-Modify versus Repeat trials with a plural output successfully generalized to trials with a singular output form (Fig. 4*B*), demonstrating that the decoded neural signals reflect morphological inflection itself, not phonological modification.

#### Morphological inflection does not require existing lexical representations

Having established that morphological inflection is supported by a shared, abstract neural mechanism across English and Spanish in noncognate real words, we next investigated whether lexical overlap enhances the sharedness of inflectional processing and whether lexical representations are even necessary for engaging the mechanism at all.

Cognates, which share both form and meaning across languages, offer a test of whether shared inflectional effects become more aligned when lexical entries are also shared. If so, this would suggest that lexical overlap strengthens the expression of shared inflection. On the other hand, pseudowords, which lack any lexical representation, offered the most stringent test on the universality of the inflection mechanisms by testing whether it can operate independently of lexical status or language membership.

**Figure 5.**
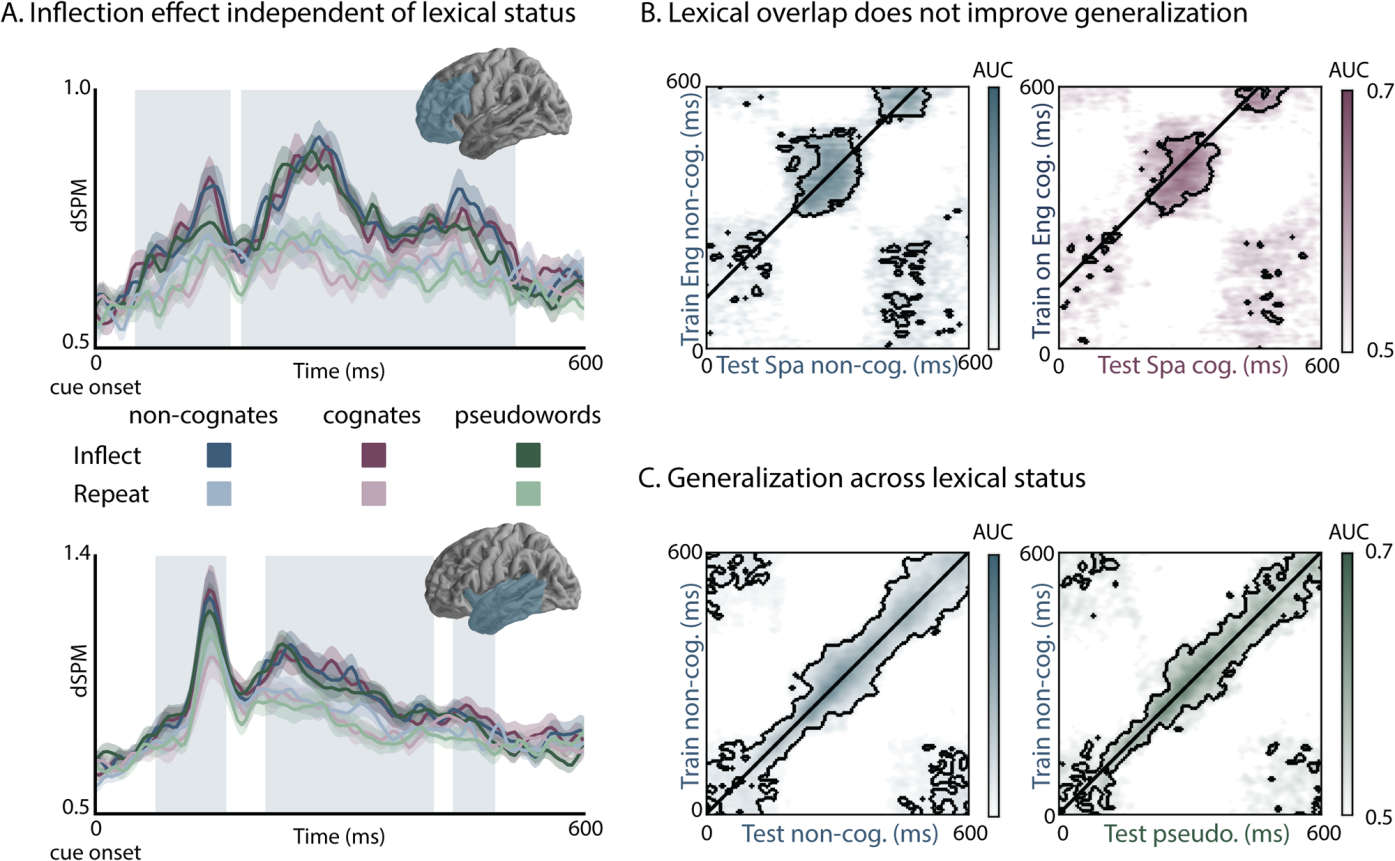
***A***, Inflection effect independent of lexical status. Brain schematics indicate the ROIs in which the univariate analyses were conducted. Line plots show the time course of activity (dSPM) for Inflect and Repeat trials across noncognates, cognates, and pseudowords. Significant time windows at *p* < 0.05 are shaded. Inflection elicited robust responses in both ROIs for all three word types, demonstrating that the core inflectional response does not depend on lexical status or stored word representations. ***B***, Lexical overlap does not improve generalization. Temporal generalization matrices show cross-language decoding when classifiers were trained on English noncognates and tested on Spanish noncognates (left) or trained on English cognates and tested on Spanish cognates (right). The solid line marks the optimal train–test temporal alignment for decoding accuracy; solid outlines encompass significant decoding points. The absence of enhanced or earlier decoding for cognates indicates that shared lexical entries do not strengthen cross-language generalization. ***C***, Generalization across lexical status. Temporal generalization matrices display decoding performance when classifiers were trained on noncognates and tested on noncognates (left) or pseudowords (right). The diagonal line marks matching training and testing times; solid outlines indicate clusters of significant decoding.

To examine this, we focused on trials involving the +s plural marker, which is also the form used in pseudoword pluralization. We conducted a temporal clustering ANOVA within the ROIs identified as supporting inflection, with Inflection (Inflect vs Repeat), Language (English vs Spanish), and Word Type (Noncognate, Cognate, Pseudoword) as factors. The results showed no significant Inflection × Word Type interaction, indicating that inflectional processing does not differ across cognates, noncognates, or pseudowords. Instead, we found robust effects of inflection in the frontal (50–165 ms and 180–515 ms, *p*s < 0.001) and temporal lobes (75–160 ms, *p* < 0.001; 210–415 ms, *p* < 0.001; and 440–490 ms, *p* = 0.008) across all word types (Fig. 5*A*). Importantly, pseudowords alone elicited reliable inflection effects (frontal, 65–115 ms, *p* = 0.014; 215–385 ms, *p* < 0.001; 395–475 ms, *p* < 0.001; temporal, 100–145 ms, *p* = 0.010; 200–270 ms, *p* < 0.001; 295–390 ms, *p* < 0.001), demonstrating that lexical representations are not required to engage the inflectional system.

Decoding analyses aligned with this pattern. If lexical overlap strengthened cross-language generalization, cognates should have shown enhanced or earlier cross-decoding, given that they share both form and meaning across English and Spanish. However, cross-language decoding for cognates was indistinguishable from noncognates (Fig. 5*B*), indicating that shared lexical entries do not provide a privileged route into the inflectional system. Critically, this lexical independence extended beyond real words: classifiers trained on inflection patterns in real, noncognate, words generalized robustly to pseudowords (Fig. 5*C*). Because pseudowords lack any lexical representation, successful generalization to them demonstrates that the neural signature of inflection does not rely on stored lexical entries and can operate over entirely novel forms.

Together, these results extend the core finding that the left frontotemporal mechanism for morphological inflection is abstract enough to generalize not only across different surface forms and languages, as shown in earlier analyses, but also to pseudowords which lack lexical entries entirely. While cognates do not uniquely enhance inflectional overlap, the overall consistency across word types underscores the lexicon-independent and language-general nature of the underlying inflectional machinery.

## Discussion

This study provides compelling evidence that morphological inflection engages a shared, abstract neural mechanism across languages, surface forms, and word types. Using a combination of univariate and decoding analyses, we demonstrate that this mechanism operates independently of surface phonology, lexical overlap, and even lexical representation, offering new insights into how bilingual brains process morphology and challenging long-standing assumptions in both psycholinguistics and neurolinguistics.

### Shared morphological mechanisms across languages

A central and enduring question in bilingualism research is whether morphosyntactic processing is language-specific or shared (Hartsuiker and Pickering, 2008; Kroll et al., 2015). Our findings clearly support the shared architecture view, demonstrating that morphological inflection in both English and Spanish recruits a largely overlapping left-lateralized frontotemporal network, consistent with prior evidence for cross-linguistic morphosyntactic convergence (Tolentino and Tokowicz, 2011; Hartsuiker and Bernolet, 2017; Declerck et al., 2020). Critically, this overlap extended to noncognate words, ruling out explanations based on lexical or phonological similarity and instead reinforcing the hypothesis that bilinguals maintain a shared abstract grammatical system (Hartsuiker et al., 2004), at least at the level of inflectional morphology.

Importantly, our results extend this principle of sharedness beyond syntax to encompass the core mechanisms of morphological inflection. The observed overlap and generalization across languages demonstrates that shared neural processing is not limited to higher-order sentence structure but also governs word-level morphosyntactic transformations. These findings align closely with language nonselective models in which morphosyntactic operations apply across languages in parallel, even in the absence of surface similarity or shared lexical entries. In particular, our data provide empirical support for the Integrated Language System model proposed by Blanco-Elorrieta and Caramazza (2021), which argues against modular, language-specific architectures and instead posits a fully unified system where lexical, syntactic, morphological, and phonological representations are shared across a bilingual's languages.

Recent work by Declerck et al. (2020) further supports this integrated perspective. They observed a bilingual sentence superiority effect when processing grammatical mixed language sentences, suggesting that bilinguals rely on a common syntactic architecture. This insight is directly mirrored in our findings: just as syntax appears to operate over a shared structural store, we show that inflectional processes, often thought to be more tightly coupled to surface forms, are likewise abstracted from language membership and phonological realization. In a recent EEG study using word reading, Cho and Brennan (2025) showed successful cross-language decoding of the number between Korean and English. Their finding provided strong evidence that certain morphosyntactic features are shared across languages, despite drastic differences in typology and surface forms.

Finally, while the spatial profile of inflectional activity was consistent across languages, our results revealed a systematic ∼110 ms temporal offset, with Spanish trials eliciting earlier activation of the inflectional network than English. This timing difference closely matches the behavioral RT advantage for Spanish and reflects processing efficiency rather than distinct neural mechanisms. One possible source of this offset lies in subtle differences in morphological regularity and production dynamics across the two languages. Although plural marking was surface-aligned in our design, Spanish pluralization is highly regular and nearly exceptionless, whereas English maintains a broader plural system that includes irregular forms and phonologically conditioned allomorphy. Even when only regular items are tested, English plural production may require additional resolution between stored lexical alternatives and rule-based computation or later phonological specification of allomorphic variants (/s/, /z/, /ɪz/), whereas Spanish plural formation proceeds via a highly uniform morphophonological mapping. Such differences could delay the onset of inflectional encoding in English. These patterns therefore point to a shared but flexibly timed processing stream, adapted to the morphological properties of each language.

Taken together, across languages, plural types, word types, and task structures, results reinforce the view that bilinguals possess a language-general, abstract morphological system, in line with the findings of a universal language network across different language families and typologies (Malik-Moraleda et al., 2022). Our findings contribute to a broader theoretical shift in bilingualism research: away from models that isolate grammatical processing by language and toward models that treat morphosyntactic computation as shared, dynamic, and structurally unified across languages.

### Inflection is separate from phonological change

A long-standing concern in morphology research is whether inflectional effects merely reflect phonological modification, such as segmental addition (e.g., *cat*→*cats*). However, our data clearly dissociate inflection from phonological modification. Both inflections that involved phonological changes and those that did not (Inflect-Modify vs Inflect-NoModify) elicited overlapping neural signatures.

Moreover, we found no evidence that addition versus subtraction of phonemes (e.g., pluralization vs singularization) affected the underlying neural processing mechanism, despite some prior accounts proposing asymmetries in processing costs for additive versus subtractive morphological operations at least in irregular inflections (Stockall and Marantz, 2006). We did observe greater activation for the production of singular inflected forms, which may reflect markedness asymmetries in morphological systems, where singulars often represent the default but cognitively more demanding option in certain derivational contexts.

### Lexical independence of the inflectional system

A striking finding of this study is that morphological inflection operates robustly even in the absence of lexical representations, as shown by the successful inflection of pseudowords. These results challenge models that tightly couple morphological operations to lexical access (Levelt et al., 1999) and instead support accounts that treat inflection as a rule-based, productive process that can apply generatively to novel inputs (Pinker, 1991; Taft, 2004). Importantly, pseudowords elicited reliable inflectional effects in both frontal and temporal regions and generalized in decoding analyses trained on real words, demonstrating that inflectional mechanisms generalize across both known and novel items.

The successful decoding of pseudowords, using classifiers trained on real words, confirms that inflectional mechanisms are lexicon-independent, a hallmark of a fully productive morphological system (Halle and Marantz, 1994). These findings contribute to long-standing debates about zero morphology and inflectional underspecification (Embick and Marantz, 2008). Our evidence that both overt and covert (zero-marked) plural forms engage the same neural circuits supports the view that zero morphemes are morphosyntactic entities, not phonological absences (Bobaljik, 2000). Furthermore, the success of cross-decoding between overt and covert markers suggests that the abstract inflectional process is the shared computational core, regardless of surface realization.

### Cognates do not enhance, and pseudowords do not impair

We further tested whether lexical overlap enhances the degree of inflectional sharing across languages by including cognates, which share both form and meaning across languages. While one might expect such overlap to increase temporal or spatial synchrony across languages, we found no evidence for enhanced overlap of inflectional effects in cognates relative to noncognates. This finding aligns with the broader result that inflectional processing does not require shared lexical entries and adds nuance to the role of cognates in bilingual processing (Costa et al., 2000; Dijkstra and van Heuven, 2002). Rather than modulating the core inflectional process, cognates may primarily affect lexical access and selection, leaving the downstream morphological computation unaffected. Importantly, prior demonstrations of cognate facilitation and lexical boost effects typically arise in tasks sensitive to lexical access or surface-form coactivation. In contrast, the present decoding analyses were trained to discriminate Inflect versus Repeat trials while collapsing across lexical identity. The classifier therefore targeted the neural signature of performing a morphosyntactic transformation and under this framework, lexical overlap would enhance decoding only if lexical representations contributed directly to the inflectional neural code. The absence of a cognate boost thus suggests that the representational format supporting inflection operates at a level abstracted away from surface form. This interpretation is consistent with recent neuropsychological findings showing that cognate status does not confer protection from language impairment (Blanco-Elorrieta and Arantzeta, 2025). Such results suggest that similar surface form alone does not guarantee a privileged representational status across languages.

In contrast, the finding that pseudowords elicit full inflectional responses reveals the remarkable generativity and abstraction of the bilingual inflectional system. The fact that inflectional effects were robust across real and novel items supports the view that the bilingual brain possesses a language-general, form-driven rule system, consistent with theories of morphological computation that emphasize productivity, abstraction, and domain-general learning.

### Conclusion

In summary, the current study identified a shared neural mechanism for morphological inflection in bilinguals and provided support for models of bilingual language system that call for an integrated and unified language system incorporating elements and rules of both languages. The current study narrowed in on a perfectly matched case where the two languages share the same rules and forms for the morphological inflection tested. Future research should extend on the current finding and further explore whether the sharedness of the morphological system could be extended to languages that drastically differ in the richness of their morphological systems.
